# An Agent-Based Model for Tertiary Educational Choices in Italy

**DOI:** 10.1007/s11162-021-09666-4

**Published:** 2021-12-14

**Authors:** Silvia Leoni

**Affiliations:** University of Leicester, Business School Leicester, Leicester, UK

**Keywords:** Agent-based modeling, Higher education, Italy, C63, I23, D31

## Abstract

Although the low level of tuition fees and the absence of other access barriers, Italy is characterized by low educational attainments at the university level. This work models the choice of young Italians to attend university or leave education and enter the labor market, by making use of an agent-based model that reproduces the Italian higher education and policy system. The aim is to analyze the determinants behind university enrollment decisions possibly causing the low level of attainment and explore three alternative scenarios that propose the expansion of financial support and the increase in the average income gap between skilled and unskilled individuals. The model implies that the individual preference to enroll at university depends upon (i) economic motivations, represented by the expectations on future income, which are formed through interaction within individuals’ social network; (ii) influence from peers; (iii) effort of obtaining a university degree. Results show that the model can reproduce observable features of the Italian system, and highlights low income levels and the following full resort to regional scholarships. Experimented scenarios show that policies expanding financial support to education are ineffective, while an increase in the gap between average income of skilled and unskilled workers leads to an increase in enrollment in university, signaling that labor market policies may be more effective than educational policies in raising the number of students in higher education.

## Introduction

Despite low tuition fees and the absence of other entry barriers, Italy is characterized by low levels of attainment at the tertiary education level. Within the 10-year European strategy Europe 2020, the European Commission elaborated five targets to be reached by 2020, among which the goal concerning education included reaching a share of 40% of 30–34-year-old completing a third level education. These common goals translated into national targets for member states. Italy, in particular, aimed at reaching 26–27% of people aged between 30 and 34, having attained a tertiary education degree. Indeed, according to Eurostat, in 2017 the 26.5% of Italians aged between 30 and 34 had a higher education (HE) degree, placing Italy in line with national targets. Nevertheless, Italy performs badly when compared to the other EU countries: a lower share is registered only in Romania (26.3%).

According to the OECD, the Italian tertiary education system is not attractive to potential students (OECD, 2016). This is demonstrated by the low entry rate to tertiary education and the high share of the so-called NEETs (i.e., people who are Not in Education, Employment or Training) which increased over the period 2009–2019 (OECD, 2020). 37% of Italians are expected to enter a bachelor’s program for the first time before they turn 25, with respect to the OECD average of 45% (OECD, 2019). Viesti (2018) suggests that low university enrollment rates in Italy could be explained by a growing distrust of Italian families towards investment in higher education, which may be affected, among other factors, by low earning levels of young graduates when entering the job market.

Moreover, Naticchioni et al. (2016) who investigated earnings gaps across generations in Italy, found that the generation born between 1975 and 1979 suffered from a remarkable earnings loss from the first entry in the job market, with respect to the previous generations. In particular, high-skilled workers of the 1975–1979 generation, who completed a tertiary educational level, have been affected by a decrease in their earnings in a much larger measure than low-educated workers, without a catch up of this gap in the following years. Franzini and Raitano (2019) studied inequality in earnings in Italy and showed that the increasing wage inequality over the last two decades is not driven by higher skill premia. Wages appear widely dispersed across tertiary graduates, signaling high riskiness of the investment in education.

This paper introduces a model of how individual educational preferences form, with a double purpose. First, it aims at analyzing the determinants behind university enrollment decisions and exploring whether these determinants could explain the low educational attainment characterizing Italy. The model implies that the individual preference to enroll at university depends upon economic motivations, influence from peers, and the effort of obtaining a university degree.

Second, the model explores the effect of three different scenarios: (a) an increase in the income cap for obtaining a partial fee waiver; (b) the introduction of a No Tax Area exempting from tuition fee payment; (c) an increase of the average income gap between skilled and unskilled individuals.

The novelty of this contribution mainly resides in the methodology; the model is explored by means of agent-based computational simulations. Agent-based models allow to take into account heterogeneity of agents, social interaction, and exchange of information, and through their “bottom-up” approach they permit to explain macro-consequences starting from micro-processes. This approach is particularly suited to study complex phenomena, where emergent results are not simply the aggregation of single agents’ behaviors, and to experiment scenarios that it would not be possible to test in the real world. From a theoretical point of view, the methodology employed also contributes to overcoming the limitations posed by standard literature, allowing for the analysis of educational decisions with a multidimensional approach.

The paper is organized as follows. "Agent-Based Models for Educational Decisions" section reviews the literature on agent-based models adopted for studying educational decisions. "The Higher Education Policy Context in Italy" section provides an overview of the Italian higher education policy context. "The Model" section describes the theoretical model and "Simulating the Model" section explains the process of calibration, imputation, and the simulation algorithm. "Results" section presents the computational results obtained through simulations and "Sensitivity Analysis" section reports the results of the sensitivity experiment. Finally, conclusions are drawn in "Final Discussion" section.

## Agent-Based Models for Educational Decisions

This work contributes to the literature in an original way by means of the methodology adopted, that is agent-based modeling.

According to Salgado and Gilbert (2013) agent-based modeling can be defined as “a computational method that enables researchers to create, analyse, and experiment with models composed of autonomous and heterogeneous agents that interact within an environment, in order to identify the mechanisms that bring about some macroscopic phenomenon of interest". Thus, an agent-based model is typically characterized by three elements (Macal & North, 2010):

1. A set of autonomous *agents*, owning attributes and rules of behavior, able to act on their own in response to the events happening, and to take decisions independently. Agents can also be heterogeneous, meaning that their characteristics and behaviors can differ in definition and extent. Heterogeneity can exist at the set-up of the model but it may also result from the process of interaction, further differentiating agents with respect to the model initialization.
2. *Interaction* among agents and between agents and the environment, defined by a set of rules governing relationships. Through these interactions, agents obtain local information.
3. The *environment*: agents also interact with the environment which can be solely a social environment, i.e. a map of the relationships among agents, or could represent the spatial location of agents.

Agent-based modeling is employed in a variety of fields, which have in common the aim to study phenomena in their complexity. In the social sciences, agent-based modeling is particularly suited for studying large-scale outcomes deriving from the interaction among individuals. These interactions produce emergent results, that cannot be deduced by simply aggregating the properties of the agents. Through assumptions, rules, and computer simulations, agent-based models allow studying emergent properties arising from the micro-processes among agents (Tesfatsion, 2006).

This approach is effective for the purpose of this paper for three main reasons. First, it allows to consider a greater level of complexity, i.e. considering a wider range of aspects and characteristics which would be difficult to include in more standard or econometric settings and which make the model closer to the real world. Second, it allows to model social interaction and thus influence from other agents and the environment, which, as in the real world, affect educational choices. Third, it permits to experiment with potential policies, as well as testing assumptions without having to rely on data availability.

The application of agent-based modeling in the field of economics is nowadays pretty established, however very few studies have adopted this methodology to study schooling decisions and issues related to the economics of education. What this study draws from the previous literature adopting agent-based models is their usefulness for studying microeconomic policy issues and for describing society from a micro perspective.

Henrickson (2002) studied college choice matching with college acceptance in the USA, by considering the concept of cultural capital (see Bourdieu, 1984). Reardon et al. (2018) used agent-based modeling to study to what extent socioeconomic status-based affirmative action in college admissions can produce racial diversity in the USA. Applications, admissions, and enrollment in American colleges are also simulated in Fu (2014), and Reardon et al. (2016) who use an agent-based model to explore how socioeconomic inequality determines stratification.

Previous studies about education in Europe include Grow and Van Bavel (2015), who study assortative mating and gender inequality in education through an agent-based model, and Friant (2015), who uses simulation to assess the impact of school choice on school segregation in French-speaking Belgium.

The study most closely related to this work is Manzo (2013). Using empirical data for France from the year 2003, Manzo (2013) shows that among the determinants of the distribution of educational choices across social groups, social influence among agents of a network cannot be ignored. Ability and subjective perceptions of education benefits are not sufficient on their own to generate the actual stratification of educational choices across educational backgrounds existing in France at the beginning of the twenty-first century.

This work has many points in common with the study of Manzo (2013), however, it departs from his work and contributes to advance the literature on the topic of educational choices in three ways. First, it provides a new modeling framework, which is adapted to the Italian context but it could also provide a basis for studying different countries or educational systems if calibrated with appropriate data. Second, it specifically focuses on tertiary education, rather than on the general transition to a higher educational level. Educational choices at the tertiary level, in fact, might be determined by different drivers with respect to lower educational levels, when factors such as parents’ role or proximity to school could have a larger impact. Third, the model presented investigates the effect of policy tools aimed at increasing the enrollment rate and, thus, it could represent a framework for modeling policy implementation.

## The Higher Education Policy Context in Italy

When enrolling in higher education for the first time, Italian students may benefit from scholarships and financial support which in general are based on income or merit requirements, but they may also cover a variety of very specific cases (e.g. fee reduction in the case of siblings enrolled in institutions located in the same region, or in case of disability of the student herself or her parents, or in case of enrollment in a STEM discipline, etc.). If income-based, total or partial fee exemptions requirements are related to the level of equivalent economic situation indicator (ISEE), an indicator for the economic situation of households. In 2017, the Italian government introduced the No Tax Area which applies to all national HE institutions and allows those with an ISEE below €13,000 not to pay university tuition fees and ensures fee reductions for those with an ISEE between €13,000 and €30,000. Income cap may differ among universities and be larger than the minimum requirement established by law. For instance, the University of Pisa, in Tuscany, has recently approved an increase in the threshold for the No Tax Area from €23,000 to €26,000, which makes it the Italian HE institution with the highest threshold. The income requirement is the only requirement to access the No Tax Area at the first year of enrollment, but, in the following years, it is combined with merit criteria that require a certain number of academic credits to be obtained within set deadlines.

Universities may also grant partial or total fee exemptions on the basis of merit only in the first year of enrollment. This benefit may vary among universities as no general rule exists. A common case is a full fee waiver to which students are entitled if they have obtained the maximum grade at the completion of secondary school, which equals 100 or 100 *cum laude*, on a scale that in Italy ranges from 60 as the lowest grade to 100 *cum laude*.

These financial benefits are directly offered by universities at the moment of enrollment, but another common financial support is provided by regions. Regional bodies devoted to study rights (e.g. LazioDiSCo in Lazio, Erdis in the Marche region) publish an annual call for applications which establishes the income requirements (and merit requirements in years of enrollment following to the first year) to obtain a yearly scholarship which may include a cash sum, daily meals at the university restaurant, free accommodation in a student dorm or a contribution for rent payment in the case of students relocating for studying. Being eligible for a grant also entitles students to total exemption from university fees. However, not all those eligible actually receive the scholarship as the number of applicants may not meet the amount of regional financial resources. Nevertheless, the national resources offered to regions have increased over the latest years (149 million € in 2013; 217 million € in 2017) and so did the share of scholarship recipients over eligible students (74.9% in the a.y. 2013/14; 97.6% in the a.y. 2019/20).^1^

The reception of one benefit excludes being awarded with other forms of support, however, students can apply to more than one type of financial aid and if eligible for multiple cases, they will be granted with the major benefit.

Turning to student loans remains an unused practice in Italy, not least because the state cannot pay out the amount directly but can only act as a guarantor. Generally, government-subsidized student loans are available in many European countries, however, their use is limited with respect to grants and it varies widely among countries. For example, more than 50% of first cycle students use them in the UK, but less than 1% in Italy (Eurydice, 2020).

## The Model

The model is populated by two generational groups of agents:

- *Junior*—have completed secondary education and have to decide whether to enroll or not at university;
- *Senior*—are in the labor market and can be skilled or unskilled, depending on whether they attended university in their previous period.

Agents are organized in a social environment constituted by *N* neighborhoods representing the agent’s social relations (family and friends). This environment is modeled as a social network, structured in social circles as proposed by Hamill and Gilbert (2009). This network structure is able to embed key characteristics of real social networks and exploits the circle tool: its circumference will contain all those points within a set *social reach*, i.e. radius, and creates a cut-off, limiting the size of personal network. Following Hamill and Gilbert (2009), agents included in one’s circle given a certain social reach will be the agent’s neighbors only if they can reciprocate, i.e. agents are only permitted to link with others whose reach includes themselves.

Juniors will ponder the choice to enroll at university only if they satisfy a budget constraint determined by (i) a monetary endowment deriving from their parental income, and (ii) the cost of education, which can be reduced or annulled if students obtain a scholarship or a waiver.

Juniors satisfying the budget constraint will enroll at university with a probability $Pr_{it}(enr)$ defined as a non-linear function increasing in the level of preference $P_{it}$ which is an additive function of three elements:

1. Economic motivation, represented by the real expected consumption on the time $t+1$ which considers expectations on future income. These are defined on the basis of interaction with senior agents in the neighborhood (set by the social reach);
2. Social influence, i.e. the influence from peers, reflecting "educational conformism";
3. Effort, i.e. the disutility for obtaining a university degree, which depends on the level of *ability*, *a*, ranging between 0 and 1, owned by each agent and capturing innate talent and personal skills.^2^

In each period of the simulation, juniors satisfying the budget constraint, observe the income of senior neighbors and educational choices of their peers, and set their preference for enrolling. They then make a decision over continuing their studies at the tertiary level and eventually become senior agents. If they opt for starting to work immediately, they will begin earning an income drawn from an unskilled income distribution; if they enroll at university they will be assigned with a skilled income once they have completed their degree. The model assumes no unemployment and immediate access to the labor market after studies. Students in higher education can also drop out and become unskilled seniors.

In each period, a portion of junior and senior agents respectively enters and leaves the model in a process of birth and death ensuring population dynamics, but that does not intent to mimic real demographics.

The following sections describe the model in detail.

### Budget Constraint

The budget constraint faced by junior agents is the following:

1 $$\begin{aligned} X_{i,t} - CostEdu >0, \end{aligned}$$

where $X_{i,t}$ represents the endowment agents are provided with; *CostEdu* is the cost of education, which is assumed to be exogenous and fixed according to the educational system considered.

If the budget constraint is not satisfied, junior agents will directly enter the job market as unskilled, without evaluating the possibility to enroll in HE.

The endowment $X_{i,t}$ is computed as a share of parental income; this value is proxied by the average propensity to save of Italian households computed by Istat (Italian National Institute of Statistics) and fixed to 8% for the last quarter of 2019. This choice comes from the nature of the variable endowment: it can be considered as a sort of bequest, rather than a consumption entry.

*CostEdu* is set on the basis of data from the 2017 report of Federconsumatori on the expenses linked to Italian universities.^3^ The average estimates for Italian resident and non-resident university students per year are listed in Table 1.

**Table 1:** Average yearly expenditure for Italian university students, for different income ranges and, if non-resident, for renting a single or a shared room

	Non-resident students	Resident students
II income range		1425.63
In single room	9415.7	
In shared room	7944.1	
III income range		1668.82
In single room	9658.89	
In shared room	8187.29	

Source: Federconsumatori

Income ranges are defined on the basis of the ISEE level. II range includes ISEE equal or less than 10,000€ while the III range includes ISEE up to 20,000€

Values reported in Table 1 include costs for accommodation (only for non-resident students), bills, transportation, tuition fees, handbooks, and study material. Starting from these values, the cost of education has been approximated to 5000€ per year and multiplied by 3 to consider the full length in years of an undergraduate degree.

### Consumption

If the budget constraint (1) is satisfied, then real expected consumption for skilled workers (*s*) at time $t+1$ will be:

2 $$\begin{aligned} C_{i s,t+1}^e=X_{i,t}- CostEdu +Y_{i s,t+1}^{e} \end{aligned}$$

while real consumption for unskilled workers (u) will be:

3 $$\begin{aligned} C_{i u,t+1}^e=X_{i,t} +Y_{i u,t+1}^{e} \end{aligned}$$

where $Y_{i s,t+1}^{e}$ and $Y_{i u,t+1}^{e}$ are respectively the average expected income for skilled and unskilled workers. Expectations on future income are modeled as naive expectations based on the information set of senior neighbors, i.e. junior agents observe their senior neighbors’ income and simply take the average:

4 $$\begin{aligned} Y_{i,t+1}^{e}=E_{t}(Y_{t+1}|\Omega _{n,t})=\frac{\sum _{i=1}^{n v} v Y_{n Sen,t}}{n v}, \end{aligned}$$

where *n* stands for neighbors and *nSen* for senior neighbors.

In the Italian context, students’ attainment level at university is found to be highly influenced by the educational level of parents and family background in general (Bratti et al., 2008; Checchi, 2003), which influence also the probability to drop out (Cingano et al., 2007). Therefore, to consider family effect and model the influence that parents have on their children’s educational choices, agents’ connections with parents are allowed to count up to double a standard connection. This is implemented by assigning a level of importance $v = 1$ to each connection; links with parents, instead, are equal to $v = 1+r$ where *r* is a number drawn from *U*(0, 1), meaning that parental effect can count to a maximum of 2.

Given (2) and (3), agents will compare the expected consumption in the two cases by taking the natural logarithm of their ratio:

5 $$\begin{aligned} ln \bigg (\frac{C_{i s,t+1}^e}{C_{i u,t+1}^e}\bigg )=ln \bigg (\frac{X_{i,t}- CostEdu +Y_{i s,t+1}^{e}}{X_{i,t} +Y_{i u,t+1}^{e}}\bigg ). \end{aligned}$$

Each senior agent is assigned a certain level of income, randomly drawn from a distribution that is assumed to be lognormal.^4^

02 The lognormal distribution is a typical example of heavy-tailed distribution and it has been widely employed in economics. Aitchison and Brown (1954, 1957) observed that the lognormal type of distribution is particularly appropriate for the distribution of incomes, especially for the bulk of earnings from a homogeneous section of the workforce. Clementi and Gallegati (2005), using micro-data from Survey on Household Income and Wealth (SHIW) for the years 1977–2002, found that the central body of Italian income distribution is consistent with a lognormal model. Using annual data from SHIW, for the years 2002–2016, each dataset is split in two, separately analyzing the income distribution for skilled individuals, identified as those individuals owning a university degree of any kind, and unskilled individuals, those not holding a tertiary education degree.

The fit is restricted only to earnings from employees,^5^ to be consistent with what was observed by Aitchison and Brown (1954, 1957), dropping observation for self-employed.^6^ To avoid biased estimates, the work considers the sampling weights provided by the survey and whose adoption is recommended by the Bank of Italy.

For each dataset and for each year considered, the two-parameter lognormal distribution is fitted by maximum likelihood, obtaining estimates for the parameters $\mu$ and $\sigma$ for the distribution of skilled and the distribution of unskilled workers, over the eight years considered.

Given that incomes are compared across years, data are converted to 2010 prices to remove the inflation effect, using the Istat household consumption deflator. All estimates can be found in Appendix.

To account for changes over time, parameters were then averaged, obtaining the results displayed in Table 2.

**Table 2:** Average parameters estimates for a lognormal distribution of Italian incomes for skilled and unskilled individuals over the years 2002–2016; data provided by the Bank of Italy (SHIW)

	${\hat{\mu }}$		${\hat{\sigma }}$	
	S	U	S	U
2002	10.27	9.67	0.79	0.84
2004	10.20	9.62	0.89	0.90
2006	10.13	9.63	0.89	0.83
2008	10.04	9.53	0.88	0.87
2010	10.09	9.48	0.79	0.99
2012	9.51	9.18	0.79	0.95
2014	9.59	9.24	0.96	1.00
2016	9.90	9.34	0.84	1.01
Mean	9.97	9.46	0.85	0.92

Therefore, a certain level of income is randomly extracted for skilled and unskilled workers, from two distributions of the same type, identified by different parameters.

The individual income is then multiplied by the average number of expected years of working life, fixed to 32 following Eurostat.^7^

### Scholarship and Fee-Waivers

To replicate real features of the Italian university system, the model allows for three possible economic benefits which directly affect the value of *CostEdu* both in the definition of the budget constraint (see "Budget Constraint" section) and in the definition of real expected consumption for skilled agents (see "Consumption" section). Agents can only receive one type of benefit, therefore, those entitled to more than one type will receive the most convenient, that is the subsidy granting the largest amount of scholarship/reduction among the three.

*Scholarship* When parental income $Y_{pi,t}$ does not exceed a certain threshold *c*:

6 $$\begin{aligned} Y_{pi,t} < c \end{aligned}$$

students are eligible for a regional scholarship which guarantees fee exemption and covers the whole cost of education. To reflect the funding capacity of granting bodies, we set a threshold of 90% as the maximum number of grants awarded to students qualifying for the scholarship, i.e. 10% of eligible students will not obtain the grant.^8^

For students awarded with the scholarship, the cost of education will be totally funded by the government:

7 $$\begin{aligned} Costedu=0 \end{aligned}$$

To define the income threshold for the scholarship granted by the government, calls for scholarships published by the regional bodies in charge of study rights have been consulted. Coherently with the average threshold observed in the calls, the yearly ISEE threshold level for scholarship has been fixed to 22,000€. The ISEE is usually computed by centers specialized in tax assistance as it includes a variety of particular cases and its calculation is not easy. To simplify, ISEE can be roughly calculated as follows:

$${\text{ISEE}} = {{\left\{ {{\text{Household}}\,{\text{income + }}\left[ {\left( {{\text{Mobile}}\,{\text{assets + Real}}\,{\text{estates}}\,{\text{assets}}} \right){\text{0}}{\text{.20}}} \right]} \right\}} \mathord{\left/ {\vphantom {{\left\{ {{\text{Household}}\,{\text{income + }}\left[ {\left( {{\text{Mobile}}\,{\text{assets + Real}}\,{\text{estates}}\,{\text{assets}}} \right){\text{0}}{\text{.20}}} \right]} \right\}} {{\text{parameter}}\,{\text{of}}\,{\text{the}}\,{\text{equivalence}}\,{\text{scale}}}}} \right. \kern-\nulldelimiterspace} {{\text{parameter}}\,{\text{of}}\,{\text{the}}\,{\text{equivalence}}\,{\text{scale}}}}$$

In this case, any form of assets is neglected and income is assumed as the only source of wealth. The parameter of equivalence takes a value varying on the basis of the number of individuals composing the household. In our case, families are made of two agents (a parent and a child, see "Initialization and Calibration" section), so the parameter is set to the corresponding value of 1.57. It is then possible to derive the relative income value for the threshold, considering the projection over 32 years of life expectancy, and obtain a corresponding threshold level for the endowment $X_{i,t}$ assigned to junior agents, which is equal to 88,422€.

*Partial fee-waiver* A partial waiver is granted by the university when parental income $Y_{pi,t}$ does not exceed a threshold *d*, with $d>c$. In this case, students obtain a 50% fee reduction, so that:

8 $$\begin{aligned} Costedu=Total\, Costedu - (Tuiton\, fee / 2 ) \end{aligned}$$

Upon observation of Italian universities’ websites, the threshold *d* is set to an ISEE level of 30,000€, corresponding to an endowment value of 120,576€, following the reasoning applied to set the threshold *c*. Assuming an average annual tuition fee equal to 1500€, and considering the 3-year duration of an undergraduate program, the partial waiver awarded is equal to 2250€.

*Full fee-waiver* Total exemption from tuition fees is offered directly by universities on the basis of merit. Students are entitled to receive a full waiver if they have obtained the highest grade (100 or 100 *cum laude*) at secondary school completion. In this case, the total cost of education will be reduced by an amount equal to the entire tuition fee:

9 $$\begin{aligned} Costedu=Total Costedu - Tuition\, fee \end{aligned}$$

Since the final grade obtained at secondary school completion has been adopted to define ability (see "Preference for Enrolling" section), the requirement to obtain a full fee-waiver, equal to the full amount of tuition fees for the 3 years of studies (4500€), is given by ability $a=1$.

### Preference for Enrolling

*Juniors* elaborate their preference for enrolling as an additive function depending on three factors: (i) a *materialistic* term represented by expectations on consumption, which represent the economic motivation; (ii) a *social* term $SI_{it}$ and (iii) an *intangible* term, given by the disutility for the effort of education:

10 $$\begin{aligned} P_{it}=ln \bigg ( \frac{C_{i s,t+1}^e}{C_{i u,t+1}^e}\bigg )+SI_{it}-EF \end{aligned}$$

The first term, specified in (5), represents the economic motivation driving the preference for enrollment. Taking the natural logarithm of the ratio between the two types of consumption allows to approximately parameterize the resulting number in the range $(-1,1)$.

$SI_{it}$ represents *social influence*, given by the fraction of the number of peers within one’s neighborhood deciding to enroll over the total number of peers in the neighborhood. Peers are agents in a young age which have faced the educational choice in previous periods:

11 $$\begin{aligned} SI_{it}=\frac{N Peers Enr}{N_{n Peers}}, \end{aligned}$$

The formalization of the *SI* term implies that the higher the proportion of choices for continuing studying at the tertiary level, the larger the impact on the agent’s probability of also enrolling in HE. By construction, values of SI will range from 0 to 1. SI can be considered as a measure of “educational conformism” which reflects a merely imitative behavior. As explained in Manzo (2013), this type of behavior has three sides: (i) on a cognitive level, the larger SI, the more predominant in cognitive terms the tertiary educational level becomes (Harding et al., 2010); (ii) from the normative point of view, the larger the proportion of the agent’s peers deciding to enroll in HE, the higher the psychological costs in terms of individual and social identity the agent would have to bear in case of different choice (Akerlof, 1997; Akerlof & Kranton, 2002); (iii) in relation to opportunity, the larger SI, the higher the probability that the agent will have access to information and resources, such as study materials and notes, housing and transportation, during her university career, if agent decides to enroll like her peers.

The third term of equation (10), *EF*, stands for the effort necessary to obtain a university degree. It is assumed to depend on individual ability through the following function:

12 $$\begin{aligned} EF= (1-a_{it})^{\gamma } \end{aligned}$$

where $a_{it}$ measures individual ability and $\gamma >0$ measures the concavity of the function: the larger is one’s ability, the lower is the effort necessary to obtain a university degree. In particular, depending on whether $\gamma$ takes values lower, equal or larger than 1, this parameter signals the presence of *returns to scale*:

- when $\gamma <1$ returns to scale are *decreasing*, meaning that with larger levels of ability, the effort decreases less than proportionally;
- when $\gamma =1$ returns to scale are *constant*, which means that when ability increases, the effort proportionally decreases;
- when $\gamma >1$ returns to scale are *increasing*, i.e. the larger the value of ability, the lower the effort, with a more than proportional decrease.

The model is initialized with increasing returns to scale, with $\gamma =1.2$. As in Staffolani and Valentini (2007), $a_{it}$ is assumed to be included between 0 and 1; therefore, the term *EF* will assume values in the range (0, 1). The level of ability attributed to each agent is calibrated using the distribution of the final grade at secondary school completion, provided by Miur for the year 2019.^9^ According to the data adopted, 96% of students were admitted to *maturità*, which is the final exam that, if passed, guarantees secondary school attainment and access to tertiary level education. Among those admitted to the final exam, Miur provides the grade distribution displayed in Table 3.^10^

**Table 3:** Secondary school grade distribution in 2019

Grade	%
60	7
61–70	31.4
71–80	28.7
81–90	16
91–99	9.7
100	5.6
100 cum laude	1.5

Source: Miur 2019

Starting from the grade distribution, the level of ability is proxied in such a way as to obtain a value ranging between 0 and 1. Therefore, a value of ability $< 0.6$ drawn by a uniform distribution is assigned to the 4% of the whole junior population, to represent the 4% of students not admitted to the final exam. Among those admitted, a value of ability equal to 0.6 is attributed to the 7% of them, following the 7% graduating with a grade of 60, and so on for the rest of the grade classes, combining in one category those who obtained 100 and 100 *cum laude*, which will have ability equal to 1.

Given the structure of $P_{it}$, two blocks can be identified: a *monetary* block represented by the economic motivation, and a *non-monetary* block made of the intangible and social drivers identified in the rest of the equation. This provides the possibility to explore the potential predominant role of one block over the other, by assigning a weight *w* to the two parts:

13 $$\begin{aligned} P_{it}=w \bigg ( ln \bigg ( \frac{C_{i s,t+1}^e}{C_{i u,t+1}^e}\bigg ) \bigg )+(1-w)(SI_{it}-EF) \end{aligned}$$

Based on the values assumed by the terms defining this function, the preference for enrollment can assume values in the range $(-2,2)$.

*Junior* agents enroll at university with a probability increasing in the level of preference $P_{it}$,

14 $$\begin{aligned} Pr_{enr}=f(P_{it}). \end{aligned}$$

Building on Manzo (2013), this could be written as:

15 $$\begin{aligned} Pr_{it}(enr)=\frac{exp(P_{it})}{1+exp(P_{it})} \end{aligned}$$

meaning that the decision to enroll at university is assumed to be a monotonically non-linear increasing probabilistic function in the level of preference.

## Simulating the Model

### Initialization and Calibration

The model has been implemented in NetLogo. At the set-up, 100 senior agents are created, each one with an age assigned randomly starting from 23, that, considering enrollment to a 3-year undergraduate program, it is assumed as the age of completion of higher education studies. Agents aged between 25 and 45 will *hatch* a child, that is a junior agent, according to a probability named *birth rate* fixed to 0.13.

The model is initialized with a proportion of skilled agents over unskilled ones based on the SHIW . Considering waves from 2002 to 2016 the proportion of individuals owning a university degree is averaged to 9% for the model set-up.

To take into account the social context, agents are not randomly located in the world space, but they are placed according to a certain level of initial *segregation*, so to reflect educational homophily in friendship, i.e. the possibility that individuals with similar educational level will tend to have friends and acquaintances with their same educational level (Thomas, 2019). This is modeled by exploiting the NetLogo *world*, which is a grid built on a system of coordinates. The center of this grid is the (0,0) point, meaning that the space can be ideally split vertically into two halves, separated by the y-axis. Skilled (unskilled) agents will segregate in the right (left) half of the *world* according to a segregation index $p_s \in [0,1]$. At the initialization of the model, a random number $x_i$ is assigned to each senior agent *i*, with $x_i$ drawn from a standard uniform distribution. Each agent *i* will be located according to the following rule: if $x_i$ is lower than $p_s$, agent *i* is assigned to her area of segregation, if $x_i$ is higher than $p_s$, agent *i* tosses a coin to decide to which side she will be assigned:

16 $$\begin{aligned} {\left\{ \begin{array}{ll} p_i =1 &{} \text {if} \; x_i < p_s \\ p_i = \frac{1}{2} &{} \text {otherwise} \end{array}\right. }, \end{aligned}$$

where $p_i$ is the probability that agent *i* is assigned to her area of segregation.

Therefore the probability that agent *i* is assigned to her area of segregation is equal to:^11^

17 $$\begin{aligned} p_i=\frac{1}{2}+\frac{1}{2}p_s. \end{aligned}$$

The higher $p_s$, the higher the probability that a skilled agent is placed on the right side, and that an unskilled agent is placed on the left side.^12^ Like for the level of ability, it is not trivial to ground the measure of segregation to observable values. Nevertheless, sociological literature has demonstrated that homophily, including educational homophily, improves the likelihood of integration in personal networks (Marsden, 1988; Louch, 2000). We assume a setup value of 0.5 as would be for a fair coin and let other values be tested in the sensitivity analysis.

To ensure reciprocity in the social circles’ network structure, agents have all the same level of social reach fixed to 10. Distance is measured from the center of the *patch* where the agent is located, and its units represent the number of *patches*.^13^ This means that, given a certain agent, a social reach equal to 10 implies that all agents located within a radius of 10 patches from the agent if reference will be her neighbors. Therefore, just as in real life, the size of the personal network will be different for every agent.

When juniors are *hatched*, they inherit by default the same location of their parent, thus when they are just born, they are placed on the same patch as their parent. In order to differentiate the neighbors of parent and child, juniors have to move away from their parent agent of a certain number of steps that is fixed to 5. In this case, as well, the spatial units are represented by patches of the NetLogo world.

The model also considers the possibility for students to drop out and leave university. Italy is historically characterized by this phenomenon, whose determinants have been widely analyzed in literature (see for example Cipollone & Cingano, 2007). In each period of the simulation, a set share of students equal to 15% (taken as the average value from Miur data elaborated by ANVUR, Italian National Agency for the Evaluation of the University and Research Systems) drops out and become unskilled senior. Drop-out rate could be further investigated by endogenizing its process within the model, however, this is beyond the purpose of this paper.

Table 4 summarizes the inputs used for the model set-up.

**Table 4:** Variables’ values used in the model set-up with data source and year of reference in parentheses

Variable	Inputs and calibration
N. senior agents	100
N. steps	5
Social reach	10
Index of segregation $p_s$	0.5
Proportion skilled/unskilled	9% (SHIW Bank of Italy)
Endowment	8% (Istat)
Cost of education	5000€ per year (Federconsumatori 2017)
Regions funding capacity	90%
Average working life	32 years (Eurostat 2016)
Ability	Secondary school grade (Miur 2019)
Drop out rate	15%
Income distribution	Skilled $\sim Lognormal (9.97,0.85)$
	Unskilled $\sim Lognormal (9.46,0.92)$

### Model Dynamics

The previous section describes what happens to the model at its initialization, that is at $t=0$, where *t* represents the model time units. The model is then run for 100 periods of time. Starting from $t=1$, junior agents aged 20,^14^ which satisfy the budget constraint (1), considering the possible economic benefit according to equations (7), (9) or (8), observe their neighbours’ income and shape their expectations on future earnings as in Eq. (4).

Consequently, agents form their expectations on future consumption according to (5), compute their effort (12) and social influence (11). The latter is computed by observing the educational choices of neighbors aged between 21 and 25. Eventually, their preference for enrolling is computed. These agents will then formalize their decision to enroll or not at university, following (15). At that point, juniors deciding not to enroll in higher education “grow up" and turn their generational status into *senior*. Juniors enrolling in higher education will spend three years at university. After the 3-year legal duration of an undergraduate program, they will also change their generational status in *senior*. When agents turn senior, they are assigned with a level of income drawn from the corresponding distribution: the income distribution for skilled if they completed their studies; the income distribution for unskilled, if they decided not to study at university. These two distributions are those described in Section  Consumption and identified by the parameters reported in Table 2. In addition, when turning senior, agents relocate in the NetLogo *world* according to the segregation level implemented in the model set-up. This process happens at each *t*, which corresponds to 1 year; moving from *t* to $t+1$, the age of agents is updated consequently.

At each *t*, junior agents are born according to a certain probability, as explained in "Initialization and Calibration" section. Like what happens in the model initialization, juniors move 5 steps away from their parent in order to differentiate their personal network. In addition, at each *t*, senior agents aged more than 50 “die", i.e. they leave the model forever. In order to smooth the population dynamics a carrying capacity set to 800 is attributed to the *world*, meaning that the population can count 800 individuals at maximum. At each *t*, in case of population in excess, a number of agents equal to the excess leaves the model.

This dynamic of the population does not aim at mirroring the processes related to population happening in real life, yet in this model, it is necessary to account for factors such as parental effect and social network influence.

To understand the model behavior and give robustness to its results, a Monte Carlo experiment is performed, by simulating the model 100 times. Simulations were run using Behavior Space, which is a software tool integrated into NetLogo that allows exploring the model’s parameter space; it runs the model with each possible combination of parameters values set by the modeler and generates a dataset with results when the runs are over.

Box 2.1 summarizes the steps of the simulation algorithm programmed to collect the outputs of interest through simulations.

**Table:** Box 2.1. The simulation algorithm

1. Initialization	
(a) Population and agents’ attributes	
Create 100 *senior* agents	
Set location according to segregation	
Set seniors’ variables: age, income, educational status	
Let seniors *hatch* a junior according to birth rate	
Set juniors’ variables: age, endowment	
Let juniors move 5 steps away from parents	
(b) Environment	
Set social reach 10 to identify neighbors	
Set importance *v* for agents and parents	
2. Model dynamics	
Iterate the model 100 t. For each *t*:	
(a) Juniors aged 20 compute possible scholarship/fee-waiver. If satisfying the budget constraint:	
Interact with neighbors and compute:	
Expectations on future income	
Expectations on future consumption	
Social influence	
Effort	
Preference for enrolling	
Take a decision:	
Enroll^15^/do not enroll according to $Pr_{enroll}$	
Change generational status into senior	
Obtain a skilled/unskilled income	
Set location according to segregation	
(b) Seniors *hatch* and die according to birth rate and carrying capacity	
(c) Set-up new juniors as in the initialization procedure	
(d) Update agents’ age by adding 1 year	
(e) 15% of students in higher education drop out and become unskilled senior	
(f) Compute and update output variables	
3. Monte carlo experiment	
(a) Define output variables of which results must be collected	
(b) Run 100 experiments using the Behavior Space	

## Results

The main output of interest is given by the enrolling rate, which is computed as the percentage of juniors aged 20 deciding to enroll over the total number of juniors of the same age.

The second output of interest is represented by the share of the agents enrolling at university coming from an educated or uneducated family, thus how many have a skilled or unskilled parent, in relative terms.

Finally, the model investigates the relative number of students awarded with an economic benefit over the total number of agents enrolling at university, and the composition of benefits awarded. Box 2.2 reports an overview of the model mechanism and its outputs.

**Table:** Box 2.2. Overview of the model

Input	Income	Proportion S/U	Segregation
	Cost of education	Personal network	Family effect
	Scholarship	Fee waivers	
Model	Economic motivation	Social influence	Effort
Output	% enrolling	% Enrol. from S	
		% Enrol. from U	
	% Awarded	% Scholarship	
		% Full fee waiver	
		% Partial fee waiver	

### The Baseline Model

Table 5 lists the mean values and corresponding standard deviation of the variables of interest across the Monte Carlo simulations performed, and it compares them with observed values averaged for the period 2012–2017 or 2013–2017, depending on data availability.^16^ Values for the variables of interest are also displayed over the simulations time span, in order to compare Monte Carlo simulations and track their dynamics over the time span.

**Table 5:** Average values and corresponding standard deviations across 100 simulations, calculated over the time span 20–100, and real-world data derived from Istat, Almalaurea and Miur and averaged for the period 2012–2017 or 2013–2017, depending on availability

	Simulated results			Real world data
	Mean	sd		Mean (2012/2013–2017)
Enrolling rate %	59.32	1.00	Rate of transfer to HE %	52.2
Enrolling from skilled %	43.53	1.84	At least one parent with HE degree %	27.8
Enrolling from unskilled %	56.47	1.84	Parents with no HE degree %	70.8
Scholarship %	89.55	0.55	Graduates benefiting from a scholarship %	22.5
Partial fee waiver %	9.43	0.55	Students with partial fee waiver %	8.54
Full fee waiver %	1.02	9.21	Students with full fee waiver %	14.5

The rate of transfer to HE represents transfer from secondary school to university and it is the average value over the years 2012–2017, as provided by Istat. The shares of students with at least on parent with a HE degree or no HE degree at all refer to graduates from bachelor programs and are average values over the years 2012–2017 derived by data provided by Almalaurea. The share of graduates benefiting of a scholarship also comes from Almalaurea as average value over the years 2012–2017 and refers to graduates from undergraduate programs only. Average values for students with partial and full fee waiver refer to the years 2013–2017 and are derived from Miur based on figures for all students enrolled in public HE and including all cases ensuring tuition fee exemption

**Fig. 1:**
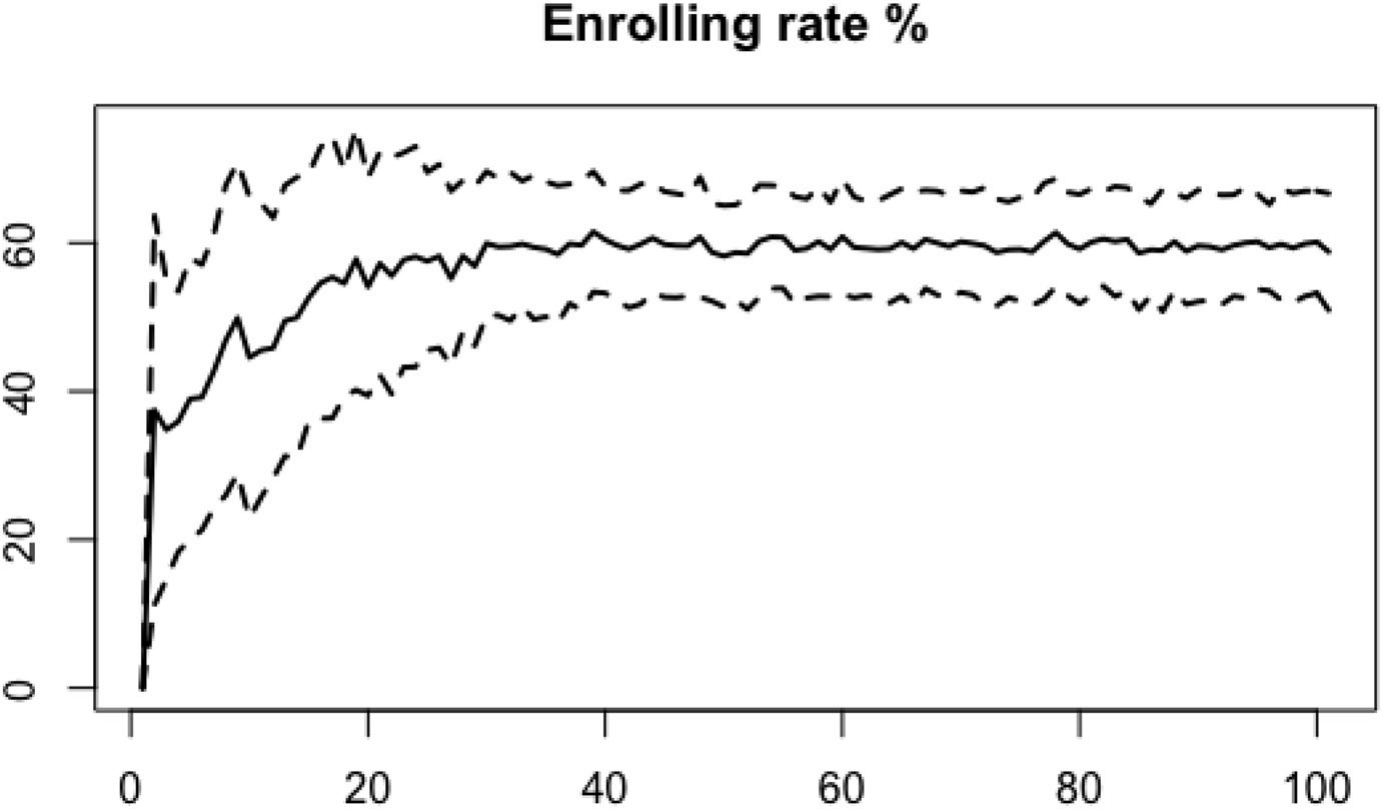
Average enrolling rate in percentage (*continuous line*) and standard deviation (*dashed line*) over the simulations time span (benchmark model)

**Fig. 2:**
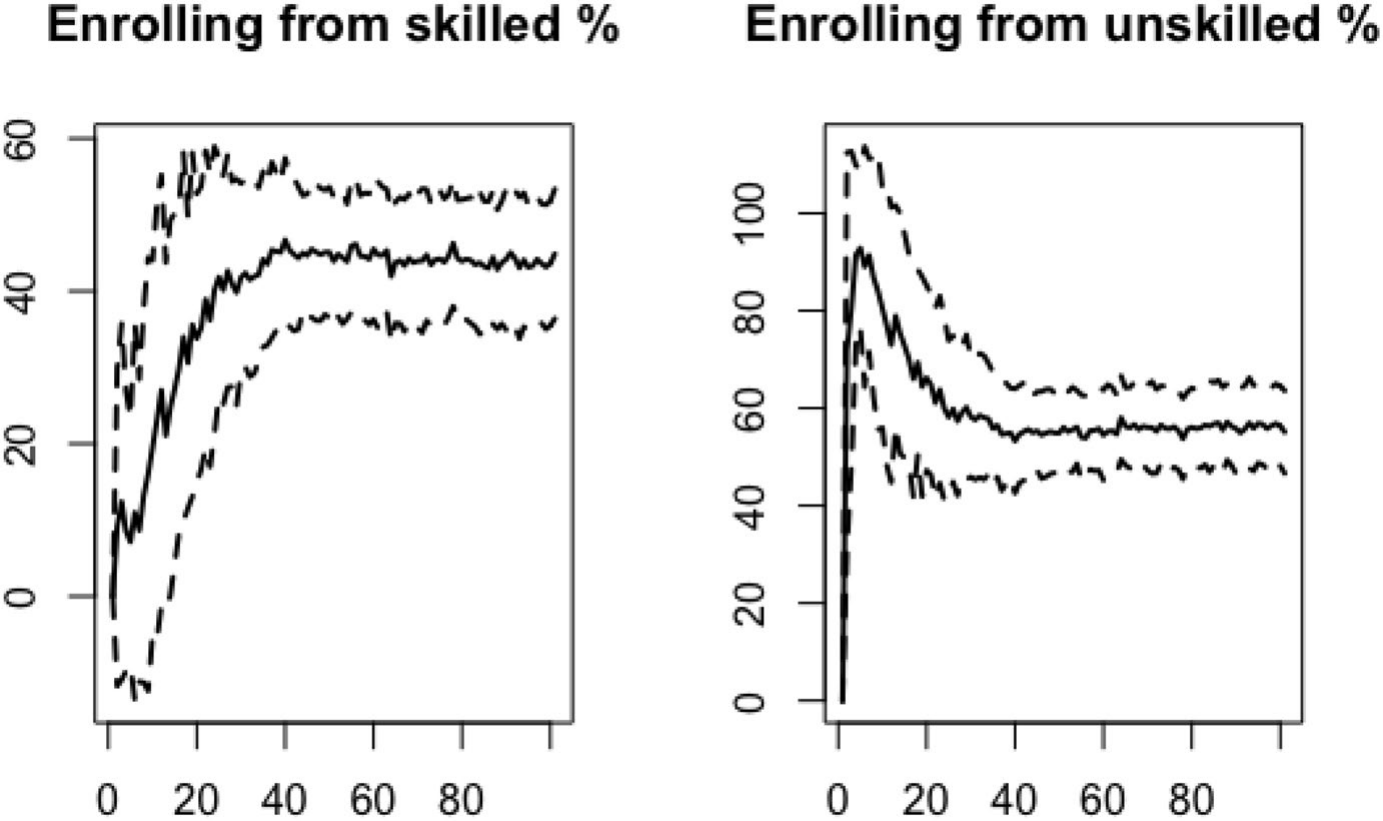
Average rate (%) of agents enrolling to university, coming from a skilled/unskilled family (*continuous line*) and corresponding standard deviation (*dashed line*) over the simulations time span (benchmark model)

The enrolling rate stabilizes around 59% (see Fig. 1) and among agents enrolling at the university, the majority comes from uneducated families (Fig. 2). These figures are in line with average values observed from real word statistics. The enrolling rate well represents the average rate of transfer from secondary school to HE as reported by Istat for the years 2012–2017 (52.2%). Computational results also reproduce the observable fact that the majority of those who completed an undergraduate program comes from unskilled families, although values are far from a precise correspondence with the real-world average values over the period 2012–2017, as reported by Almalaurea (see Table 5).^17^ However, the comparison itself could be more accurate if data on students enrolled in their first year rather than graduates becomes available.

All agents who decide to enroll at university receive some form of benefit. The majority of students receive a regional study grant (89%), coherently with the maximum financial coverage of regional authorities included in the model. The merit-based benefit, i.e. the full fee waiver, is the least used form of benefit (see Table 5). This indicates that a large share of students has a starting income that falls within the ceiling set for the other two types of benefits. These extreme results may point out, on the one hand, a low average income level: ISEE levels are below the threshold set for obtaining a regional scholarship, which is then awarded up to the full funding capacity of regions and, covering the full cost of education, leaves a little role to play to other forms of financial support. On the other hand, results may indicate low wage premia for skilled workers, so that unskilled families with lower wages benefit from financial support whereas skilled families, with higher wages, remain behind in obtaining financial aid but, at the same time, do not dispose of high enough income to support the education of their juniors. However, simulated results for financial aids show a considerable gap with average real-world figures. In particular, this is the case for the share of students receiving a scholarship, which is equal to 22.5% as observed by Almalaurea. This comparison is not the most accurate as data from Almalaurea refer to bachelor graduates, however, the remarkable gap size may imply that the regional funding system, which plays a key role in supporting studies in Italy, may need to be shaped more precisely and as an additional set of autonomous agents, which also takes into account the financial availability of regional authorities. Simulated results for agents benefiting from a partial fee waiver (9.43%) are similar to what observed by Miur (8.54%) for the years 2013–2017, while adherence to reality is less for those who obtain a full fee waiver: the simulated average result is 1.02% against 14.5% average value derived by Miur. However, the 14.5% value inflates the real figure since it considers all students enrolled in any type of degree in all public universities and it includes any possible case in which the tuition fee was not requested, including exclusion due to student impairment and awarding or eligibility for the regional scholarship which also represent cases of exemption from tuition fee payment.

### Alternative Scenarios

Given the context defined by the baseline model, we present three alternative scenarios which simulate two policy experiments and an abstract framework.

1. We propose an increase in the ISEE threshold for obtaining a partial fee reduction, from 30,000€ to 40,000€, leaving unchanged all the other characteristics of the model, thus those eligible will be required to pay half of tuition fee to enroll in an undergraduate program. Numerous universities allow a partial waiver for a bigger threshold than the legal requirement of 30,000€, however, they often rely on *bands* associated with different levels of ISEE and a different amount of fee reduction.
2. Following the example of the University of Pisa, we introduce a No Tax Area up to a limit threshold of 26,000€ in the level of ISEE. The baseline model does not include a No Tax Area since its legal threshold (13,000€) falls within the amount of ISEE allowed for obtaining a full scholarship, which, as a larger benefit, would annul its effect by model construction.
3. Following the results observed for the baseline case, the model assumes a hypothetical scenario in which the average wage differential increases. We let the mean income level of the skilled distribution increase by 10,000€.

Average results across simulations are displayed in Table 6 together with simulated results for the baseline model for comparison.

**Table 6:** Average values and corresponding standard deviations across 100 simulations, calculated over the time span 20–100, for the baseline model and the three alternative scenarios tested

	Baseline model		Scenario 1		Scenario 2		Scenario 3	
	Mean	sd	Mean	sd	Mean	sd	Mean	sd
Enrolling rate %	59.32	1.00	59.24	0.99	59.37	0.93	65.67	0.85
Enrolling from skilled %	43.53	1.84	43.47	1.65	43.37	1.72	47.27	1.52
Enrolling from unskilled %	56.47	1.84	56.53	1.65	56.63	1.72	52.73	1.52
Scholarship %	89.55	0.55	89.59	0.54	89.57	0.59	89.59	0.55
Partial fee waiver %	9.43	0.55	9.41	0.51	0	0	9.41	0.56
Full fee waiver %	1.02	0.21	1.00	0.21	0	0	1.00	0.19
No tax area %	–	–	–	–	10.43	0.59	–	–

Results show that the two policy interventions (scenarios 1 and 2) do not alter the results of the baseline model and thus they are not effective in producing an increase in enrollment into tertiary education. Conversely, the third scenario proposing an ideal increase in the average income level of skilled workers brings the enrolling rate to 65%. This last measure proves also to act as a stimulus for enrollment by students coming from skilled families, which decide to continue their studies in a larger share (47.27%) than in the baseline model (43.53%), going in the direction of greater balance between the two educational background. This result allows us to draw two considerations. The first is coherence with research by Naticchioni et al. (2016), Franzini and Raitano (2019) and Viesti (2018), that is the fact that the gap between skilled and unskilled incomes has narrowed in recent years and that investment in university studies is not attractive to young Italians. In fact, when the gap between the average incomes of skilled and unskilled workers increases, then this difference can drive investment in education, encouraging the choice of further studies by students who come from skilled families. The second consideration is that policies aimed at education, to the extent proposed in this paper, could be ineffective, while far more effective could be to intervene in the labor market with policies aimed at increasing wages and ensuring an adequate return to university degrees.

All those enrolling in higher education benefit from a form of financial support, as in the baseline model. The participation of the different types of aid appears stiff and does not change across the first and third scenarios analyzed. However, introducing a No Tax Area with a threshold that is higher than the threshold for a regional scholarship, changes the composition of financial support. All students who do not benefit from a full scholarship are exempted from paying tuition fees following the new policy implemented, hence no student is required to pay even a discounted fee as the former support is more convenient. Although the introduction of the No Tax Area changes the contribution that the various benefits provide to students, the greatest financial burden continues to fall on regions to the extent of their capacity, since they offer the greatest benefit. In addition, results for agents using the No Tax Area are in line with Miur data which report an average value of 9% for the years 2017–2019 (considering, however, all students enrolled in public HE).

## Sensitivity Analysis

The model is characterized by the presence of several parameters and inputs affecting each one of the three components of the preference for enrolling. To understand whether and to what extent these parameters affect the results of the model, a sensitivity analysis is carried out by performing a “parameter sweep", that is by repeating the Monte Carlo experiments for different values of the parameters. This is performed on the baseline model, as well as on the different policy scenarios proposed to investigate whether different parameters affect the magnitude of the enrollment increases resulting from policy application.

To isolate the effect of the parameters, the seed of the pseudo-random number generation process is set to 15 at the initialization of the model. This guarantees that the sequence of pseudo-random numbers will be the same in all the simulations of the sensitivity analysis, and it is important to isolate the effect of changes in the parameters’ values from the effect produced from changes in random numbers.^18^

$\gamma$ As discussed in "Preference for Enrolling" section, $\gamma > 0$ rules the concavity of the relationship between individuals’ ability and effort and indicates the presence of *returns to scale*. In addition to the value of 1.2 assigned to the parameter in the baseline model, a sensitivity experiment is performed on three additional values respectively identifying decreasing, constant, increasing returns to scale: 0.5, 1, 1.8.

*Segregation* The segregation index $p_s$ defines educational homophily. In addition to the value of 0.5, as in the benchmark model, the index is now set to 0.3 and 0.8.

*No. of steps* When they are *hatched*, junior agents walk 5 steps away from their parent’s location. In the sensitivity analysis, values equal to 10 and 15 are also considered.

*Social reach* The social reach is given by the radius of agents’ social circle. We let it vary to 5 and 20 with respect to value 10 in the benchmark model.

*Endowment* According to Istat, in the second quarter of 2020, the propensity to save of Italian households grew to 18.6%, possibly as a result of the coronavirus outbreak and lockdown policies. We investigate possible variations in the model caused by a growth of propensity to saving and, therefore, to the endowment, to 20%.

*Weights* As explained in "Preference for Enrolling" section, the model allows for weighting the tangible and intangible block composing the preference for enrolling. Hence, the model is simulated for values of the weight *w* equal to 0.1, 0.5, 0.8.

*Drop out rate* The model sensitivity is tested also for different values of drop out. We assume two alternatives situations: a null level of drop out and a double level with respect to the benchmark model, thus 30%.

*Regional funding capacity* We test two hypothetical values corresponding to a reduction of resources availability to 70% and to an unlimited capacity to award full scholarships, corresponding to 100%.

*Birth rate* Sensitivity is also tested for different values of the probability to *hatch* new agents.

**Fig. 3:**
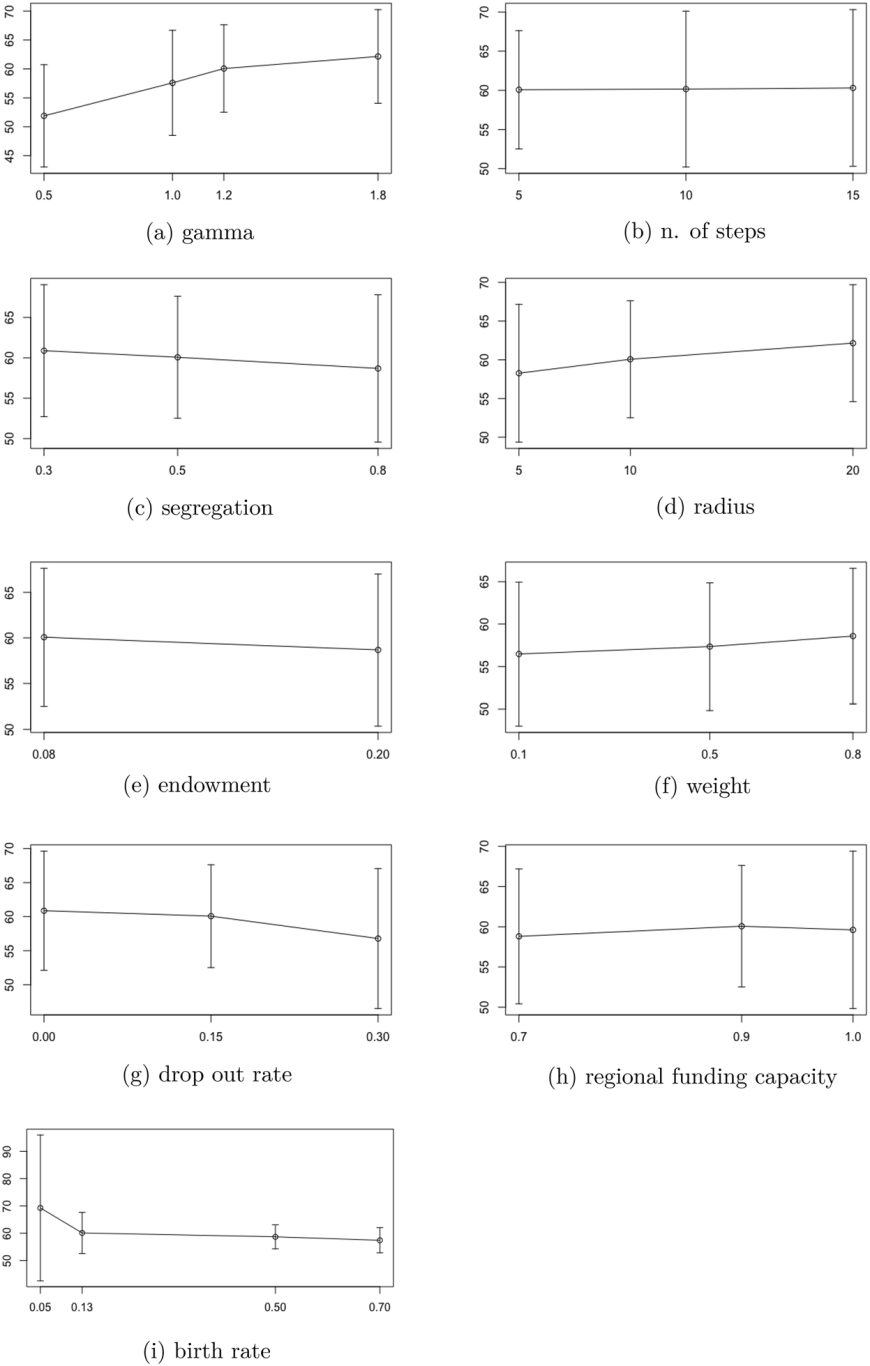
Synthetic statistics for the sensitivity experiments on the baseline model: enrolling rate

The results of the sensitivity analysis are displayed in Fig. 3 for the main output of interest, i.e. the enrolling rate, for the baseline model. The model’s reaction to parameter changes does not vary when the sensitivity analysis is applied to the three scenarios, thus the discussion applies to all cases analyzed. The figure shows the mean and standard deviation of the variables of interest in the various sensitivity scenarios.

The model appears to be sensitive to changes of the parameter $\gamma$: increasing the value of the parameter causes a slight increase of the enrolling rate with respect to the benchmark value, while its volatility is not particularly affected. This is consistent with the theoretical predictions of the model, given that for $\gamma >1$, increasing returns to scale reduce more than proportionally the disutility of pursuing a university career in terms of effort, thus stimulating the decision to enroll at the university. Consistently, when $\gamma$ takes values lower than 1, the enrolling rate shows lower levels, as for higher levels of ability the effort decreases less than proportionally, thus producing a weaker stimulus on the preference for enrolling. The overall effect appears to be stronger in the case of diminishing returns to scale.

A slight sensitivity is also shown with respect to variations of the measure of segregation. A higher probability to be located in proximity of agents with the same educational level, thus higher educational homophily in personal networks, with respect to the baseline value, produces a small reduction in the enrolling rate. Viceversa, heterophily in personal networks may help increase the enrolling rate as the latter slightly increases when the index of segregation equals 0.3. The real-world implication of this, albeit moderate, result may be that personal networks composed by people with varied socio-economic background could encourage college enrollment. Since we are considering individuals who have just completed secondary school, we may assume that these networks are largely formed by classmates. Thus, having school classes with heterogeneous backgrounds could be strategic for increasing participation in tertiary education.

The model is also sensitive to variations of the radius of the social circle; in particular, when this changes from 5 to 10, we observe an increase in the average enrollment rate, accompanied by a reduction of volatility. Thus, the enrolling rate appears to be increasing in the personal network size. This could highlight a difference in the educational choices taken by individuals with different levels of sociability, or more importantly, individuals living in rural or urban areas, as different population density would determine different school class sizes and personal network sizes. This implication could be particularly relevant for the Italian territory where differences between urban and rural areas could highlight further differences among e.g. North and South.

When weights are assigned to the determinants of preference for enrolling, the sensitivity analysis shows that a higher enrolling rate is associated with slightly higher values of *w*. Recalling equation (13), this equals saying that enrollment rate grows when a higher weight is attributed to the monetary block of equation (13), signaling that rather than social influence or effort, income might be the main driver of the preference for enrolling. In addition, when the model includes weights, the rate of enrolling is moderately smaller than in the baseline model.

Moderate changes in the results can be observed also for variations in the input value adopted for the drop out rate: when this doubles, the enrolling rate decreases coherently with the increasing number of unskilled agents populating the model.

For a small value of the birth rate, the model shows a higher level of enrollment which is likely to be the consequence of the limited population of junior agents in that case; on the other hand, for any other value tested the model is stable.

Variations in the rest of the parameters do not cause sensitive changes in the model outcome, however, it is curious to observe a slight decrease in the enrolling rate when endowment increases. On the one hand, the endowment is determinant for the formulation of the budget constraint; yet, on the other hand, it appears both at the numerator and denominator of the expectation on extra consumption (see (5)), leaving income as the main driver for the formation of extra expected consumption. Likewise, changes in the regional funding capacity do not produce significant variations in the enrolling rate: on the one hand, this highlights the importance of alternative tools of financial support coexisting with full scholarships; on the other hand, evidence shows that regional bodies for study rights manage to finance almost all eligible students.

## Final Discussion

The model developed in this work explores the factors influencing educational choices at the tertiary level and the impact of three alternative scenarios aimed to increase the level of enrollment in higher education. It takes into account both economic and social motivations, approaching the economics of education with an innovative methodology and a multidimensional perspective. The ABM approach in fact allows considering social influence deriving from interaction with a set of neighbor agents as it would happen in real life, and to account for multiple dimensions of one phenomenon, making it a useful tool to reach proximity with reality. Moreover, the novelty of the methodology for analyzing education in Italy could open a new way of studying this topic also for other countries.

*Junior* and *senior* agents populate the model, where they interact with each other. Juniors who have just obtained their secondary school diploma, are faced with the decision to continue their studies at the university or drop out and immediately enter the labor market. The probability to enroll is increasing in the level of the preference for enrolling, which depends on three main elements: economic reasons, social influence from peers, and the immaterial cost of obtaining a university degree. These terms take into account the educational segregation of agents, level of ability, eligibility for financial support and interaction. Simulations show that the average enrolling rate is about 59%, in line with actual data on the transfer rate of Italian students from secondary to tertiary education. The majority of those enrolling in higher education comes from unskilled parents (56%), as observed in the Italian context, and all students in higher education receive a form of financial benefit. This may highlight a low level of Italian incomes and its inability to pull the preference for enrolling, as well as low wage premia to skilled individuals with respect to unskilled ones, coherently with findings by Naticchioni et al. (2016) and Franzini and Raitano (2019). The financial benefits may support those with lower wages, earned by unskilled families, whereas skilled families earning higher wages are eligible for benefits to a lesser extent but do not have high enough incomes to provide tertiary education for their juniors.

We then experiment three different scenarios that depart from the baseline model by introducing two expansive policies which respectively increase the income cap to obtain a partial fee waiver and implement a No Tax Area for annual incomes up to 26,000€, as an initiative taken in place by only one Italian university; the third scenario experiments an abstract framework which sees an increase in the average wage differential between skilled and unskilled individuals. Simulations of the two scenarios proposing policies aimed at extending financial support produce similar results to the baseline model. On the contrary, the abstract scenario proposing an increase of the average income of skilled workers leads to an increase in the level of enrollment to 65% and an increase in the share of students coming from skilled families. This result supports literature findings suggesting that skilled workers’ incomes have narrowed their gap with unskilled incomes and that investing in higher education studies in Italy appears risky and unattractive. Moreover, the implication that can be drawn from this result is that policies targeted to increase education by providing further financial support may be ineffective in the measure proposed in this research, whereas labor market policies aimed at increasing wages and return to education may be more effective in raising the number of students in HE.

Experiments show that the maximum financial availability of regions is fully exploited and that, by construction, the contribution of different forms of financial support is rigid. However, when introducing a No Tax Area up to an income that is higher than the threshold established for eligibility for a regional scholarship, fee reduction becomes pointless since students will benefit from the more convenient No Tax Area ensuring full fee waiver. Nevertheless, this work has highlighted the need to model more accurately, perhaps with an additional set of agents, scholarship awards, and the financial capacity of regional bodies responsible for the right to study, whose functioning may be more complex than what is represented in this model through simplification.

A sensitivity analysis has been carried out on the parameters governing the model behavior and inputs set in the initialization procedure. Results indicate that the model is robust to changes in the values of the parameters and only show slight variations for some of the values tested. The most remarkable variations are observed for values of the parameter $\gamma$ ruling the concavity of the relation between ability and effort: consistently with the theoretical prediction of the model, the relationship between individual ability and effort to obtain a university degree presents decreasing (increasing) returns to scale for values of the parameter $\gamma$ smaller (larger) than 1. Results of this analysis also highlight the potential relevance of size and heterophily in personal networks to obtain more socially desirable levels of enrollment. Further research could explore the relationship between the network of friendships and educational decisions, by focusing on the composition of secondary school classes or providing a geographical analysis that investigates differences between e.g. urban and rural areas. The ABM approach makes this work a unique reference framework for studying the decision-making process of education and not only that. This approach allows to easily add modules, such as universities receiving students, or a geographical environment allowing for spatial analysis, and it allows to experiment abstract policies and analyze their effects before these are implemented, contrary to mainstream methods. This ABM can be directly used to further study the Italian educational system, for example investigating the antecedents of drop out or, by varying parameters and initial conditions, it could be adapted to different educational systems, allowing to analyze the decision-making process concerning education in other countries. Finally, this research revealed an important difference between simulated and real data with respect to scholarship disbursement. Hence, the importance of accurately modeling scholarship disbursement with reference to the actual financial capacity of the institutions. Implementing different forms of financial support within the model could also help study the feasibility of educational policies from the point of view of institutional spending.

## Estimations Results

This section provides the estimations results of the fit of a 2 parameter lognormal distribution to Italian personal income, as reported in Table 2. Estimations have been performed in Stata 14 using the package lognfit.

Table 7 reports the estimation of the parameters $\mu$ and $\sigma$ for all the waves of SHIW considered for the calibration of the income distribution, specifically the years 2002, 2004, 2006, 2008, 2010, 2012, 2014 and 2016. Given the necessity to refer to individuals as the unit of observation, rather than households, this work employs data provided in the annual databases. Each database has been split in two based on the education status of individuals, by means of the variable *studio*, which measures with progressive numbers (from 1 to 8) the level of the degree held by the individual, starting with no education up to a postgraduate qualification.

**Table 7:** Estimates of the parameters $\mu$ and $\sigma$ of a lognormal distribution

	Skilled			Unskilled		
	$\mu$	$\sigma$	N	$\mu$	$\sigma$	N
2002	10.27***	0.791***	1205	9.671***	0.837***	12,281
	(0.0228)	(0.0161)		(0.00739)	(0.00523)	
2004	10.20***	0.889***	1283	9.625***	0.901***	12,634
	(0.0248)	(0.0176)		(0.00802)	(0.00567)	
2006	10.13***	0.894***	1356	9.626***	0.826***	12,063
	(0.0243)	(0.0172)		(0.00752)	(0.00531)	
2008	10.04***	0.881***	1457	9.533***	0.872***	12,220
	(0.0231)	(0.0163)		(0.00789)	(0.00558)	
2010	10.09***	0.788***	1664	9.479***	0.995***	12,040
	(0.0193)	(0.0137)		(0.00907)	(0.00641)	
2012	9.513***	0.787***	1750	9.184***	0.951***	11,880
	(0.0188)	(0.0133)		(0.00873)	(0.00617)	
2014	9.591***	0.964***	1752	9.245***	0.998***	11,759
	(0.0230)	(0.0163)		(0.00921)	(0.00651)	
2016	9.896***	0.843***	1506	9.344***	1.015***	10,340
	(0.0217)	(0.0154)		(0.00998)	(0.00706)	

* p < 0.05, **p < 0.01, ***p < 0.001

## Footnotes

1 Data are retrieved from the Osservatorio Regionale per l’Università e per il Diritto allo studio universitario, based on the elaboration of data provided by Miur, the Italian Ministry of Education, University and Research.

2 Although ability is unobservable, the empirical literature has proved that regardless of the measure adopted, it affects educational decisions (see, for instance, Stocké, 2007; Cheadle, 2008).

3 Federconsumatori is a non-profit association aimed at informing and protecting consumers.

4 The probability density function of a lognormal distribution is the following:

$$\begin{aligned} p(x)= \frac{1}{x\sigma \sqrt{2 \pi }}exp\bigg [-\frac{1}{2}\bigg (\frac{log x - \mu }{\sigma }\bigg )^2\bigg ], x>0 \end{aligned}$$

where the location parameter $\mu \in {\mathbb {R}}$ and the shape parameter

02 $\sigma >0$ are the mean and the standard deviation of the normal distribution (Caiani et al., 2016).

5 Earnings are net of tax and social security contributions.

6 According to Brandolini and Cannari (1994), the SHIW underestimates self-employment income by about 50%.

7 The value is derived from the estimated duration of working life in years for a person who is 15 years old in 2017, by country, provided by Eurostat.

8 The information corresponds to the average data for the years 2012–2019 provided by Osservatorio Regionale per l’Università e il Diritto allo studio universitario.

9 The distribution of the final grade at secondary school completion is available for the year 2020 as well, however, the model uses data for 2019 to avoid possible distortions caused by the Covid-19 pandemic outbreak and the following schools’ closure.

10 Less than 1% of students failed the final exam. This percentage is considered negligible and therefore it is not included in the model.

11 Before the extraction of $x_i$ the probability that agent *i* is assigned to her area of segregation is equal to

$$\begin{aligned} p_i = 1 \cdot prob(x<p_s) + \frac{1}{2} \cdot prob(x_i >p_s), \end{aligned}$$

given that $x_i \sim U(0,1)$, it follows that $prob(x<p_s)=p_s$ and $prob(x>p_s)=1-p_s$ from which equation (17) follows.

12 Notice that if $p_s =1$ then $p_i =1$, meaning that all skilled agents are on the right, and all unskilled agents are on the left, therefore there is complete segregation. If $p_s =0$ then $p_i =\frac{1}{2}$ meaning that each agent has the same probability of being on the left or on the right, therefore there is no segregation.

13 A *patch* is a square piece of the *world* over which agents can move.

14 This age approximately corresponds to the age in which Italian students complete secondary education and potentially start their university studies.

15 In this case, the next two actions take place at $t+3$.

16 The initial period of the simulations (20-time units) is neglected as it represents a transient phase.

17 Almalaurea is an Interuniversity Consortium which surveys the profile and employment status of graduates annually.

18 A pseudo-random numbers generator (PRNG) is a deterministic algorithm producing a sequence of numbers that presents almost the same statistical features of a sequence generated by a random process. The resulting generated sequence of numbers is not truly random: it is entirely determined by a small set of initial values (usually just one) called ”seeds”(Caiani et al. 2016).
